# An Efficient Hybrid Methodology for Local Activation Waves Detection under Complex Fractionated Atrial Electrograms of Atrial Fibrillation

**DOI:** 10.3390/s22145345

**Published:** 2022-07-18

**Authors:** Diego Osorio, Aikaterini Vraka, Aurelio Quesada, Fernando Hornero, Raúl Alcaraz, José J. Rieta

**Affiliations:** 1 BioMIT.org, Electronic Engineering Department, Universitat Politecnica de Valencia, 46022 Valencia, Spain; dosorio@upv.es (D.O.); aivra@upv.es (A.V.); 2 Arrhythmia Unit, Cardiology Department, General University Hospital Consortium of Valencia, 46014 Valencia, Spain; quesada_aur@gva.es; 3 Cardiovascular Surgery Department, Hospital Clínico Universitario de Valencia, 46010 Valencia, Spain; hornero_fer@gva.es; 4 Research Group in Electronic, Biomedical and Telecommunication Engineering, University of Castilla-La Mancha, 16071 Cuenca, Spain; raul.alcaraz@uclm.es

**Keywords:** atrial fibrillation, electrogram, complex fractionated atrial electrograms, local activation waves, detection, invasive recordings

## Abstract

Local activation waves (LAWs) detection in complex fractionated atrial electrograms (CFAEs) during catheter ablation (CA) of atrial fibrillation (AF), the commonest cardiac arrhythmia, is a complicated task due to their extreme variability and heterogeneity in amplitude and morphology. There are few published works on reliable LAWs detectors, which are efficient for regular or low fractionated bipolar electrograms (EGMs) but lack satisfactory results when CFAEs are analyzed. The aim of the present work is the development of a novel optimized method for LAWs detection in CFAEs in order to assist cardiac mapping and catheter ablation (CA) guidance. The database consists of 119 bipolar EGMs classified by AF types according to Wells’ classification. The proposed method introduces an alternative Botteron’s preprocessing technique targeting the slow and small-ampitude activations. The lower band-pass filter cut-off frequency is modified to 20 Hz, and a hyperbolic tangent function is applied over CFAEs. Detection is firstly performed through an amplitude-based threshold and an escalating cycle-length (CL) analysis. Activation time is calculated at each LAW’s barycenter. Analysis is applied in five-second overlapping segments. LAWs were manually annotated by two experts and compared with algorithm-annotated LAWs. AF types I and II showed 100% accuracy and sensitivity. AF type III showed 92.77% accuracy and 95.30% sensitivity. The results of this study highlight the efficiency of the developed method in precisely detecting LAWs in CFAEs. Hence, it could be implemented on real-time mapping devices and used during CA, providing robust detection results regardless of the fractionation degree of the analyzed recordings.

## 1. Introduction

Atrial fibrillation (AF) is currently the most common cardiac arrhythmia in the Western world, showing a significant impact in the quality of life of the patients [1]. Compared to 25 years ago, AF incidence has increased by 31%, while prognostics about AF evolution are dispiriting [2]. Apart from being a life-threatening disease, AF can provoke significant healthcare costs [1]. Taking all the aforementioned into account, it is clear that an efficient AF treatment is imperative. Catheter ablation (CA) of pulmonary veins (PVs) is nowadays the first-line AF therapy, being especially beneficial for paroxysmal AF patients, since they show a high rate of PVs-only foci [3,4].

Longer time in AF, often accompanying persistent AF cases, has been associated with more severe health complications and higher AF recurrence rates [1,5,6]. The CA of areas that tend to initiate or sustain the AF activity is considered an additional step following the CA of PVs in order to increase the CA success probabilities, although showing controversial results [1,7]. Coronary sinus, superior vena cava, left atrial posterior wall and interatrial septum are some of the most frequent sites where arrhythmogenic activity has been reported [8,9,10]. The main concept of the additional CA approach is the electrical isolation of the areas that provoke or perpetuate the AF dynamics. The first step of the out-of-PVs ablation of persistent AF, consequently, consists of the detection of these areas, which form the AF substrate [11].

Two main theories exist regarding the behavior of the AF dynamics, which are each connected with a different approach to confront AF. The first theory supports the existence of meandering rotors, the ablation of which, assisted by phase mapping, is believed to terminate AF [12,13]. Nevertheless, this is considered an ambiguous method due to the possibility of passive rotors’ activity [14,15,16]. The second theory attributes AF to single and random potentials, colliding with existent anatomical conduction blocks [17]. Studies based on this theory suggest the ablation of complex fractionated atrial electrograms (CFAEs), which are thought to represent areas of anatomical irregularities. CFAEs are defined as electrograms (EGMs) with activations of ≥2 deflections and/or perturbed baseline or as EGMs with a cycle length (CL) ≤ 120 ms [18]. While CFAEs’ detection can be performed without CL estimation, many techniques suggest the CL-based CFAEs definition in order to locate candidate CA targets [19,20,21]. As CL is defined by the distance in samples between two consecutive local activation waves (LAWs) [22], this requires the prior detection of LAWs.

In unipolar EGMs, LAWs annotation, known as local activation timing annotation, is relatively simple, and many algorithms have been developed around the steepest deflection technique in order to optimize the annotation performance [23,24,25,26]. However, bipolar EGMs, which are vastly used in AF mapping, are especially sensitive to various parameters such as the wavefront direction, the electrode specifications and filtering that may affect the amplitude and morphology of the signals [27,28,29,30]. Despite the development of methods aiming to facilitate the automatic annotation of bipolar EGMs, the aforementioned parameters significantly complicate the LAWs detection in CFAEs and EGMs with a high fractionation degree [31]. Taking all the afore into account, the development of algorithms able to reliably detect LAWs from CFAEs has been a rather complicated issue.

The adaptive amplitude threshold-based method, the CL-based method and the dominant frequency method are previous techniques developed for this purpose, showing competitive results [32,33,34]. Notwithstanding, the complex nature of EGM dynamics in CFAEs leads to insufficient performance in cases of high complexity. Therefore, the elaboration of an efficient and robust algorithm that will be able to detect with high accuracy the activation waves which are propagating the AF activity is still pending. The aim of the present study is to develop a high-performance LAW detector, which is able to operate reliably even in the cases of high EGM fractionation, in order to assist the CA procedure for out-of-PVs ablations. The method developed is a combination of two already existent methods: the adaptive amplitude-based and CL-based LAWs detection [32,33]. The key factor of this method, however, is the amplification of low-amplitude components in CFAEs, by applying a hyperbolic tangent (HT) function, allowing the respective activations that in other cases would be ignored to be detected and annotated.

The remainder of the document is structured as follows. Section 2 presents the EGMs dataset used in this study, as well as the data preprocessing and classification, further including an explanation of the main algorithm and the evaluation methods used for the validation of the results, which are presented in Section 3. Section 4 analyzes the main improvements and outcomes. Finally, Section 5 highlights the most relevant aspects of the present study.

## 2. Methods

The dataset employed in this study was composed of 119 10-s EGMs obtained using a CardioLab system (General Electric, Wauwatosa, WI, USA) after written consent of 22 persistent AF patients (17 male, 49.55 ± 11.54 years old) undergoing first-time CA procedures. Two expert physicians blinded to the algorithm manually annotated the dataset and classified the EGMs by AF types according to Wells’ classification [35], among which the AF type I and III are the least and most complex EGM cases, respectively. The final database consisted of 16 AF type I, 19 AF type II and 84 AF type III EGMs. The decision of selecting a considerably higher amount of type III EGMs was motivated by the fact that type III EGMs’ and CFAEs’ automatic annotation is by far more challenging and therefore a better testing environment for the algorithm’s reliability.

### 2.1. Preprocessing

**Figure 1:**
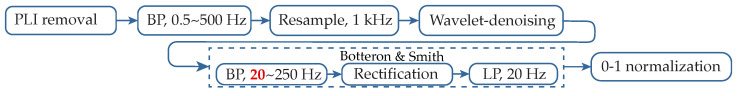
Block diagram of the preprocessing steps performed by the proposed algorithm. Botteron’s lowest cut-off frequency has been reduced to 20 Hz (red). PLI: powerline interference; BP: band-pass filter; LP: low-pass filter.

**Figure 2:**
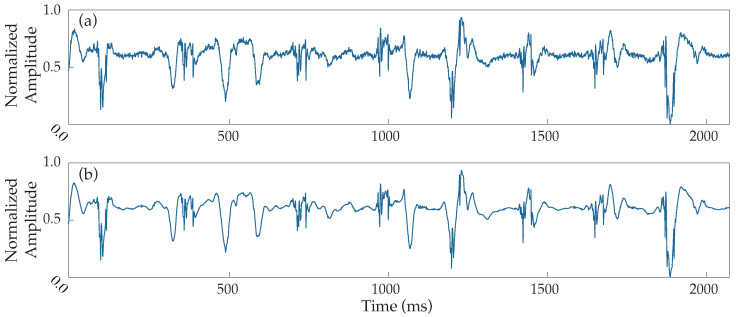
Example of noise filtering performance applied to a noisy EGM. (**a**) Normalized raw electrogram, (**b**) electrogram denoised by the stationary wavelet transform.

The block diagram of the preprocessing steps can be found in Figure 1. An adaptive notch filter to remove powerline interference, as well as a band-pass filter between 0.5 and 500 Hz to reduce the noise of low and high frequencies, were applied to the original data, which were then resampled at a rate of 1 kHz. EGMs underwent stationary wavelet transform (SWT) denoising to reduce high-frequency noise. Given the high performance of the SWT reducing noise and, at the same time preserving the waveforms’ integrity, this filtering technique is considered highly powerful [30]. Figure 2 presents an example demonstrating the performance of SWT denoising when applied over a noisy EGM.

The proposed method implements a filtering stage inspired by the preprocessing of EGMs introduced by Botteron and Smith [22], the application of which creates proportional waveforms to the amplitude of the signal’s components with frequencies between the cut-offs of the initial band-pass filter. The output signal was then rectified and low-pass filtered (see Figure 1). These two last stages have been implemented as they were originally designed, establishing the low-pass filter cut-off frequency at 20 Hz, as it has been proved to achieve an optimal performance.

Nevertheless, deep analyses of the stage associated to band-pass filtering employed by Botteron and Smith, which originally had cut-offs of 40–250 Hz, showed a good response for high-frequency components, which are typically present in the activations of type I and II AF EGMs. However, activations in CFAEs have often slow components, the waveforms of which are not proportional to the traditional Botteron’s preprocessing technique. Consequently, the detection of these components becomes significantly complex, and slow activations are in danger of being lost.

**Figure 3:**
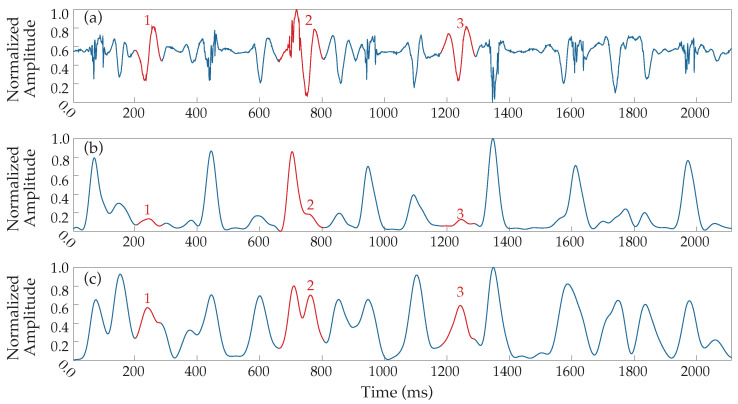
Alternative Botteron’s filtering. (**a**) Denoised EGM and resulting signals from (**b**) original Botteron’s filtering (40–250 Hz band-pass filter) and (**c**) the band-pass filter’s cut-offs modification to 20–250 Hz implemented by the proposed method. Numbers 1–3 (red) indicate three clear examples of improved responses to LAWs.

Due to that fact, multiple testing on the filtering process parameters was performed over the dataset, leading to an alternative 20–250 Hz band-pass filtering before the rectification and the low-pass filtering (see block diagram in Figure 1). The results provided a more reliable transformation, equalizing the waveform response of slow and fast activations, significantly facilitating and enhancing the detection process, and the band-pass filter was therefore implemented with this configuration. A comparative example between the outcome of the proposed signal preprocessing and the original Botteron’s filtering is shown in Figure 3. As can be observed, the new proposed wider bandwidth allows lower-frequency components to appear more fairly represented with respect to their original amplitude. Finally, signals were normalized at a 0–1 scale in order to facilitate the forthcoming analysis steps.

### 2.2. EGM Classification

**Figure 4:**
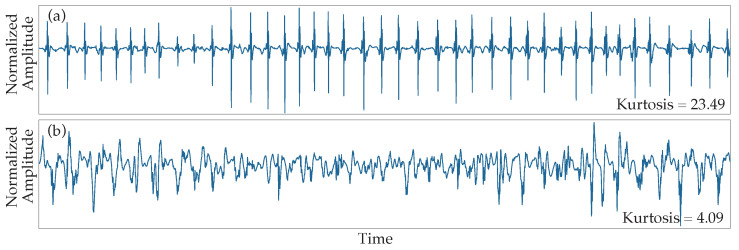
Kurtosis results to different fractionation degrees. (**a**) Type I AF EGMs normally present values of kurtosis higher than 20. (**b**) CFAEs are characterized by a low value of kurtosis. Type II AF EGMs show a wide range of intermediate values depending on each particular morphology.

The first step of the analysis consisted of the classification of EGM fractionation using the kurtosis value in one-second windows. This classification would later be necessary for the selection of CFAEs, which correspond to EGMs with very low kurtosis value, so that they can be further processed for the slow components enhancement that was described in Section 2.1. The application of the kurtosis as a fractionation index was chosen due to its simplicity and high performace in distinguishing between organized and highly fractionated EGMs, as can be seen from Figure 4.

### 2.3. Processing of CFAEs

The main issue of any amplitude-based LAWs detector is to set a reliable amplitude threshold so that a high detection performance can be achieved. Adaptive thresholds are a candidate choice in order to account for amplitude variability, which can cause significant performance issues due to abrupt changes in amplitude, which are typically present in highly fractionated EGMs. When AF types I and II are analyzed, this variability is not intense, and LAWs detection according to amplitude can lead to high precision. However, when facing the high heterogeneity of amplitudes in CFAEs, in addition to an adaptive threshold, a suitable enhancement of low-amplitude activations would be necessary. For this purpose, the HT function was applied.

**Figure 5:**
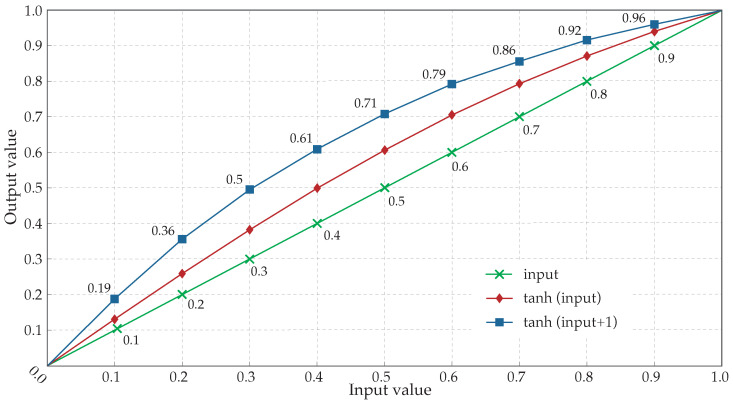
Hyperbolic tangent response to normalized input values. Diamonds correspond to the normalized output values after the application of the hyperbolic tangent to the input values (crosses). Squares refer to the response employed by the algorithm, ranging the value of the hyperbolic tangent’s argument from 1 to 2 by adding 1 to the input value. The corresponding result, once normalized, is a greater increase of the input values.

Analyses carried out showed a high response for small amplitude values and a progressive decreasing response as the amplitude values were higher. This behavior, characterized in Figure 5, provoked almost a 100% of magnification for the lowest values but, at the same time, minimum increase for the highest ones.

**Figure 6:**
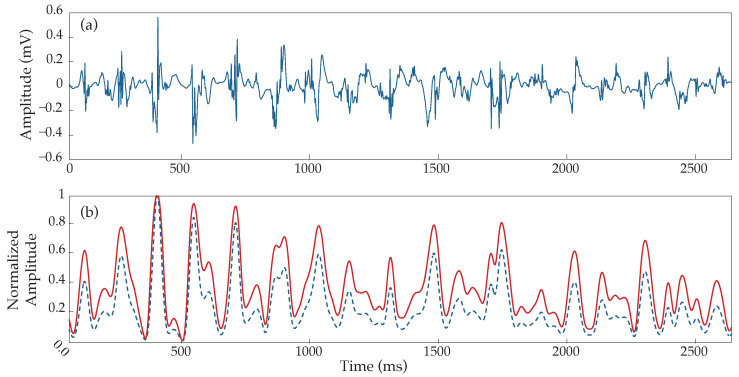
Hyperbolic tangent application over an example type III AF EGM. (**a**) Raw electrogram, (**b**) corresponding response before (blue dashed line) and after (continuous red line) the use of the hyperbolic tangent. Notice the different responses depending on the wave’s height, generating a higher increment for smaller than for higher peaks.

The application of the HT over an example signal is shown in Figure 6, where the amplitude increment can be clearly observed to be different depending on the amplitude of each activation. The result of this processing is an output signal, the activations of which are much easier to be detected using an amplitude threshold with respect to the input signal.

### 2.4. Detection Algorithm

**Figure 7:**
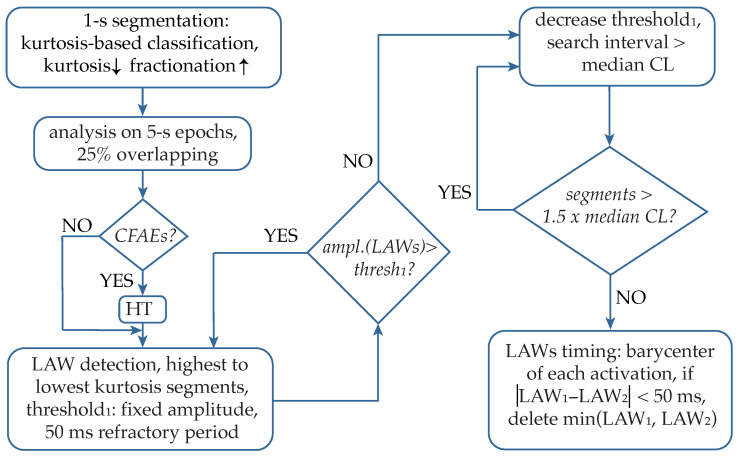
Blocks of the analysis performed by the proposed algorithm. CFAEs: complex fractionated atrial electrograms; HT hyperbolic tangent; LAWs: local activation waves; CL: cycle length.

The proposed technique is a combination of the CL-based method and the adaptive amplitude threshold-based method. The former is used as the main condition in order to keep or stop searching for new activations as well as to define the search intervals. The latter allows the progressive search of LAWs, starting with the easy cases of the most prominent activations and moving to more complicated cases, including the low-amplitude components that are present in AF type III EGMs. HT application significantly facilitates the amplitude-based detection of these low-amplitude components. Analysis was performed over five-second segments with 25% overlapping between adjacent segments, where the final LAWs detection preferentially corresponded to the segments with the lowest kurtosis value. An explanatory block diagram of the main analysis steps is illustrated in Figure 7.

Afterwards, HT was applied in low-kurtosis segments, corresponding to highly fractionated EGMs. The detection stage started using a fixed amplitude threshold heuristically established for the detection of the majority of the activations, using a 50 ms minimum refractory period (see Figure 7). Once all LAWs above the set threshold are annotated, the median CL is calculated, and the amplitude-based search is applied in intervals longer than the median CL, using an adaptive threshold that decreases according to the interval length. This process is repeated until no segments longer than 1.5× the median CL remain. Finally, for detecting activation timing (AT) of LAWs, the barycenter of each LAW is calculated as the mean value of the area of each rectified LAW. Apart from the precise localization of the AF activations, this stage allows the control of adjacently detected activations that tend to converge without respecting the minimum refractory period set. Hence, the lower amplitude activation is discarded, and the higher amplitude activation is preserved.

### 2.5. Statistical Analysis

Performance detection was evaluated by comparison with manually annotated LAWs from the physicians. Each detected activation closer than 40 ms to a manual annotation was considered as correct. Then, sensitivity, accuracy and positive predictive were calculated to provide detailed results of the method’s application on both the entire dataset and exclusively on type III AF EGMs. Specifically, with the aim to demonstrate the effectiveness of each one of the pivotal steps applied, the aforementioned evaluation was carried out after each one of them. This way, the efficacy of the induced preprocessing modification, the HT application and the barycenters calculation can be assessed.

Furthermore, accuracy was evaluated for the three different types of AF with respect to the chosen validation distance, while kurtosis’ efficacy as the CFAEs classifier was also assessed by the degree of discrepancy with the manual annotations, using a classification tree analysis with a maximum split of 2 in Matlab software (MathWorks, MA, USA).

## 3. Results

**Figure 8:**
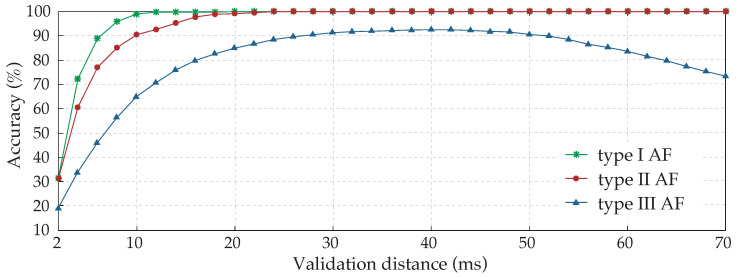
Study of the validation distance influence over annotation accuracy results for the three types of AF EGMs analyzed. Due to their complex nature, involving components of variable amplitude and frequency, type III EGMs are the most difficult to annotate automatically.

For each type of AF EGM, the evolution of the accuracy achieved by the method according to the validation distance is shown in Figure 8. As expected, type I AF EGMs reached their maximum accuracy results very soon, in a distance slightly longer than 10 ms. Type II AF EGMs showed a similar evolution, demanding a greater distance of approximately 25 ms to obtain optimal results. Lastly, a remarkable difference existed in the evolution of type III results, beginning with very poor accuracy results for short validation distances and showing a slower increase of accuracy, achieving the maximum value around 40 ms. From this point on, a progressive decrease can be observed as the distance increases, which is an exclusive behavior of CFAEs for these values due to the high degree of fractionated activity and proximity between activations.

The minimum refractory period, established in 50 ms between activations, implies that validation distances higher than this value would lead to a double correspondence between detected activations and manual annotations, provoking an accuracy reduction for AF type III EGMs, as can be observed in Figure 8. Therefore, the validation distance has to be shorter than the minimum refractory period.

Furthermore, 0.45% of the activations of type III AF EGMs were closer than 50 ms to adjacent activations, which explains the decrease in accuracy of validation distance values higher than 40 ms. This analysis would suggest the use of a shorter refractory period of 40 ms approximately, which would coincide with the same value as the validation distance. Nevertheless, results obtained with this configuration were significantly poorer due to over-detections, and this option was discarded.

**Figure 9:**
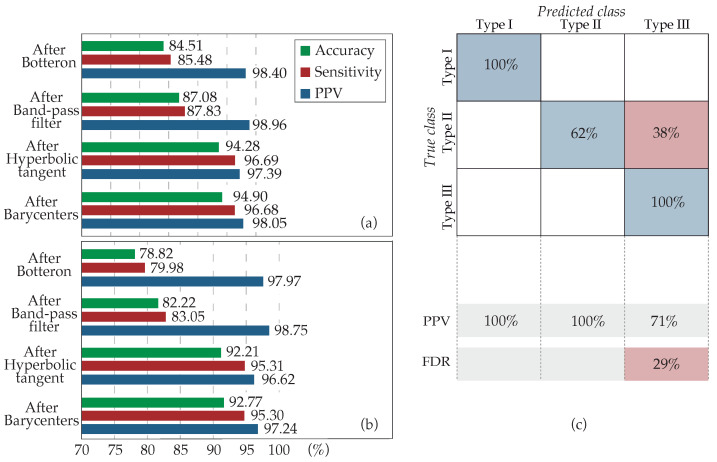
Progressive results of the method application by stages. (**a**) Over the entire dataset and (**b**) over the type III AF EGMs exclusively. Four cases are analyzed: the application of the algorithm only with the Botteron preprocessing (After Botteron), after the band-pass modification to 20–250 Hz (After Band-pass filter), adding the HT use (After Hyperbolic tangent), and, finally, with the estimation of barycenters and the deletion of close activations (After Barycenters). (**c**) Confusion matrix for the classification of the recordings according to their fractionation. True class: experts’ classification; Predicted class: kurtosis classification; PPV: positive predictive value; FDR: false discovery rate.

The overall detection performance of the method is presented in Figure 9, analyzing the application of the algorithm stage by stage. It can be observed from this figure that for both the application over the entire dataset (Figure 9a) and just over type III AF EGMs (Figure 9b), the evolution was similar. The implementation of the HT function entailed the major increment both for accuracy and sensitivity, whereas the modification of the band-pass filter cut-offs also introduced an increase but to a lesser extent. Lastly, although the estimation of the barycenter of each LAW was mainly performed for a more precise location of each activation, it added as well a slight improvement after the deletion of closely spaced barycenters, justifying its recruitment. The positive predictive value (PPV) remained almost constant in all stages, since errors made by over-detection are minimum.

Finally, Figure 9c shows the classification accuracy that kurtosis achieved for every AF type. Kurtosis has been demonstrated as a powerful estimator of fractionation, correctly classifying 100% of the AF type III EGMs. However, approximately 40% of the type II AF EGMs of the dataset, corresponding to those presenting a higher degree of fractionation or amplitude variability, were classified as CFAEs. The direct impact of this wrong AF type II classification on the results was minimal, since the application of the HT over type II AF EGMs did not introduce errors, whereas it facilitated the detection of the activations.

This evidence suggests that the HT may be applied directly over the entire dataset. However, as kurtosis is employed to monitor the evolution of the fractionation degree, its recruitment was limited to highly fractionated EGMs so that the computational cost is minimized and algorithm is optimized.

## 4. Discussion

CFAEs analysis seems to be the vulnerable spot of the already published LAWs detection methods analyzing clinical recordings [32,33,34], as performance drops significantly in this critical subgroup of AF EGMs. For this reason, various factors should be taken into account for the analysis of highly fractionated EGMs by introducing the corresponding steps. Low-amplitude and low-frequency components coexist with typical high-amplitude and high-frequency activations in CFAEs. This should be taken into consideration when CFAEs are analyzed by taking specific care of preserving and amplifying these characteristics. The present work was based on this hypothesis, applying the respective steps that have been presented in detail in Section 2.4.

Signal segmentation in five-second intervals allows the adaptability of the presented method to recordings of any length and permits the classification of EGMs by AF types at each segment individually. This way, any interval, of higher or lower fractionation than the overall fractionation level of the recording it belongs to, can be analyzed accordingly and without the bias that would exist in case of treating each recording as an entity. The choice of kurtosis as a fractionation estimator yielding fast and accurate results for the crucial AF type III EGM classification is performed so that highly fractionated EGMs, including CFAEs, can be further preprocessed with the HT function in order to enhance the low-amplitude activations that these types of EGMs often contain and increase the method accuracy, as it has been shown from Figure 9. Its application over the entire dataset, though, does not alter the obtained performance results, as it was shown in Figure 5. The reason why its application was limited to highly fractionated EGMs is the minimization of computational cost and hence, the optimization of the algorithm. Consequently, although a significant part of AF type II EGMs was classified by kurtosis as AF type III, the method’s accuracy was preserved.

The preservation of slow components is enhanced through the modification inserted in the lower cut-off frequency of the Botteron’s preprocessing band-pass filtering, being set at 20 instead of 40 Hz. This step yielded an improvement of about +2.5% in the entire dataset and about +3% in the type III EGMs, specifically. Finally, a double shield of protection against over-detections is achieved by the use of the 50 ms refractory period in the first place and the barycenter calculation, which allowed the control of converging activations into a distance less than 50 ms. Apart from providing a more detailed and precise picture of the AF substrate by optimizing the LAW timing calculation, this final step also contributed to the accuracy of the developed algorithm.

The analysis of the validation distance clearly shows the increasing discrepancy between detected activations and clinicians annotations as the AF EGM type becomes higher. Focusing on CFAEs, the optimal distance is in the range of 30–50 ms. Nevertheless, since CLs as short as 42 ms exist, a further reduction to 30–40 ms would be suggested. The setting of a validation distance as long as 30 ms, however, would lead to double detections. In order to avoid this possibility, the validation distance has finally been set at 40 ms. On one hand, Ng et al. [33] set a validation distance of 75 ms, which has been demonstrated to be excessive and would provoke double correspondences between annotations and detections in CFAEs. Furthermore, two manual annotations made by operators closer than 50 ms were not accepted in that study, which helps to avoid possible errors of under-sensing. On the other hand, Lee et al. [36] employed a maximum distance for the validation of detected activations of just 20 ms, the results of which cannot be completely validated, since the complexity of the six EGMs employed was not described.

Three of the most distinguished and established methods for LAWs detection in bipolar EGMs are the dominant frequency-based (DF) method, the CL-based method and the adaptive amplitude-based method [32,33,34]. The DF-based method detects LAWs according to the EGM’s DF, which is a method that would be unsuitable for LAW detection in CFAEs, due to the high heterogeneity of CLs, the pattern of which fails to follow loyally the DF [34]. LAW detection in the CL-based method is performed amplitude-wise until a limit CL condition is fulfilled and then seeking activations in longer segments by adapting the amplitude-threshold [33]. The main limitation of this technique is found in EGMs with highly irregular CLs or periods of sudden changes in CL. Despite the fact that high performance is achieved for normal EGMs, there exists a significant gap in CFAEs, which is the most critical scenario. Finally, the adaptive amplitude-based method adopted a threshold strategy according to the last 10 detected peaks with decreasing weights [32]. This method also failed to provide satisfactory results for highly fractionated EGMs.

The proposed method is a combination of the last two methods, the CL-based method and the adaptive amplitude-based method. By applying pivotal modifications to Botteron’s preprocessing technique as well as the HT function, this hybrid method appears to be significantly optimized even for the most critical kind of EGMs, the CFAEs. Each step has been carefully designed in order to provide higher efficiency and at the same time minimize the computational costs and simplify the procedure. Considering the high demand on detailed and precise personalized mapping on the confrontation of persistent AF and the multi-aspect high performance of the suggested algorithm, this method could be recruited for real-time AF mapping, improving CA results for out-of-PVs ablation on persistent AF patients.

## 5. Conclusions

Precise AF mapping is of paramount importance for the CA of persistent AF. The development of a robust and accurate LAWs detector regardless of the complexity of AF dynamics remains a challenge and, at the same time, an essential task for the deep knowledge about the AF substrate. The proposed method showed outstanding results even in high EGM fractionation cases, while preserving simplicity and requiring low computational resources. Analysis steps are efficient and can be reproduced easily, while signal segmentation achieves robustness and adaptability. The aforementioned advantages suggest the implementation of this technique in CA devices.

## Data Availability

The data supporting reported results and presented in this study are available on request from the corresponding author.
